# Supplementary material for: Genetically encoded assembly recorder temporally resolves cellular history
Source: Nature. 2026 Mar 3;652(8111):1049–59. doi: 10.1038/s41586-026-10323-y (PMC13102709; doi:10.1038/s41586-026-10323-y)
Supplement: Supplementary file 1 [file 41586_2026_10323_MOESM2_ESM.pdf]

nature portfolio

Corresponding author(s): Dingchang Lin

Last updated by author(s): Feb 2, 2026

Reporting Summary

Nature Portfolio wishes to improve the reproducibility of the work that we publish. This form provides structure for consistency and transparency in reporting. For further information on Nature Portfolio policies, see our Editorial Policies and the Editorial Policy Checklist.

Statistics

For all statistical analyses, confirm that the following items are present in the figure legend, table legend, main text, or Methods section.

n/a	Confirmed	
☐	☒	The exact sample size (n) for each experimental group/condition, given as a discrete number and unit of measurement
☐	☒	A statement on whether measurements were taken from distinct samples or whether the same sample was measured repeatedly
☐	☒	The statistical test(s) used AND whether they are one- or two-sided<br>*Only common tests should be described solely by name; describe more complex techniques in the Methods section.*
☒	☐	A description of all covariates tested
☒	☐	A description of any assumptions or corrections, such as tests of normality and adjustment for multiple comparisons
☐	☒	A full description of the statistical parameters including central tendency (e.g. means) or other basic estimates (e.g. regression coefficient) AND variation (e.g. standard deviation) or associated estimates of uncertainty (e.g. confidence intervals)
☐	☒	For null hypothesis testing, the test statistic (e.g. F, t, r) with confidence intervals, effect sizes, degrees of freedom and P value noted<br>*Give P values as exact values whenever suitable.*
☒	☐	For Bayesian analysis, information on the choice of priors and Markov chain Monte Carlo settings
☒	☐	For hierarchical and complex designs, identification of the appropriate level for tests and full reporting of outcomes
☒	☐	Estimates of effect sizes (e.g. Cohen's d, Pearson's r), indicating how they were calculated

Our web collection on statistics for biologists contains articles on many of the points above.

Software and code

Policy information about availability of computer code

Data collection	We used Zeiss LSM780, LSM 800, LSM980 or Observer7 for data acquisition. The associated Zen Blue or Zen Black softwares (Zeiss) were used for data collection.
Data analysis	Linear spectral unmixing was performed using Zen Blue (Zeiss). Matlab (R2023a), Python (3.11), Pymol (3.1.0) and/or ImageJ were used for other data analyses. The code involved custom scripts comprising standard image processing steps, written for the specifics of the datasets, without any novel algorithms involved. Details of data analyses can be found in the manuscripts. The code for data analysis can be found in https://github.com/DCLinLab/GEMINI

For manuscripts utilizing custom algorithms or software that are central to the research but not yet described in published literature, software must be made available to editors and reviewers. We strongly encourage code deposition in a community repository (e.g. GitHub). See the Nature Portfolio guidelines for submitting code & software for further information.

Data

Policy information about availability of data

All manuscripts must include a data availability statement. This statement should provide the following information, where applicable:

- Accession codes, unique identifiers, or web links for publicly available datasets
- A description of any restrictions on data availability
- For clinical datasets or third party data, please ensure that the statement adheres to our policy

Source datasets for in vivo experiments are provided with the paper. Complete datasets reported in this article are available at GitHub (https://github.com/LinLab/GEMINI). The plasmids constructed and used in this work are deposited to Addgene (Plasmid IDs: 228881-228886, 228888, 228889).

Research involving human participants, their data, or biological material

Policy information about studies with human participants or human data. See also policy information about sex, gender (identity/presentation), and sexual orientation and race, ethnicity and racism.

Reporting on sex and gender	N/A
Reporting on race, ethnicity, or other socially relevant groupings	N/A
Population characteristics	N/A
Recruitment	N/A
Ethics oversight	N/A

Note that full information on the approval of the study protocol must also be provided in the manuscript.

Field-specific reporting

Please select the one below that is the best fit for your research. If you are not sure, read the appropriate sections before making your selection.

☒ Life sciences ☐ Behavioural & social sciences ☐ Ecological, evolutionary & environmental sciences

For a reference copy of the document with all sections, see nature.com/documents/nr-reporting-summary-flat.pdf

Life sciences study design

All studies must disclose on these points even when the disclosure is negative.

Sample size	For each experiment, sample size was as large as could be practically achieved by our microscopic imaging, given the number of cells available in the images taken. Sample sizes were not pre-defined, but proper statistical analyses were performed to account for technical and biological variability.
Data exclusions	GEMINI particles that nucleated later than the first timestamp (without showing complete timestamps) were excluded. In seizure model, mice with a seizure score <5 were excluded. No other GEMINI particles or animals were purposely excluded from analysis.
Replication	At least three replicates are performed or include for each experiment. The reproducibility of all experiments are ensured.
Randomization	Animals were randomly assigned to experimental groups prior to data collection. For cell-based experiments, culture dishes from the same preparation batch were randomly allocated to conditions.
Blinding	Investigators were blinded to group allocations.

Reporting for specific materials, systems and methods

We require information from authors about some types of materials, experimental systems and methods used in many studies. Here, indicate whether each material, system or method listed is relevant to your study. If you are not sure if a list item applies to your research, read the appropriate section before selecting a response.

Materials & experimental systems

n/a	Involved in the study
☐	☒ Antibodies
☐	☒ Eukaryotic cell lines
☒	☐ Palaeontology and archaeology
☐	☒ Animals and other organisms
☒	☐ Clinical data
☒	☐ Dual use research of concern
☒	☐ Plants

Methods

n/a	Involved in the study
☒	☐ ChIP-seq
☒	☐ Flow cytometry
☒	☐ MRI-based neuroimaging

Antibodies

Antibodies used	anti-NeuN (GeneTex, GTX132974), anti-glial fibrillary acidic protein (anti-GFAP, Cell Signaling, #3670), anti-Phospho-IκBα antibody (Cell Signaling, 9246S), anti-IκBα antibody (Cell Signaling, 4812S), anti-Vinculin (Cell Signaling, 13901), HRP-conjugated goat anti-rabbit IgG(H+L) (ProteinTech, SA00001-2), goat anti-mouse IgG (H + L)-HRP Conjugate (Bio-Rad, 1706516), anti-CD31 (BD Biosciences, 550274), Donkey anti-Rat AF647 antibody (Invitrogen, A48272), Donkey anti-Mouse IgG (H+L) Alexa Fluor™ 488 (Invitrogen, A-21202), Donkey anti-Rabbit IgG (H+L) Alexa Fluor™ 546 (Invitrogen, A10040)
Validation	The vendors of the antibodies have provided validation, relevant test results, and/or certificates through their websites:<br>Anti-neuronal nuclear (NeuN, GeneTex, GTX132974) https://www.genetex.com/Product/Detail/NeuN-antibody/GTX132974<br>Anti-glial fibrillary acidic protein (GFAP, Cell Signaling, #3670):https://www.cellsignal.com/products/primary-antibodies/gfap-ga5-mouse-monoclonal-antibody/3670<br>Anti-Phospho-IκBα antibody (Cell Signaling, 9246S): https://www.cellsignal.com/products/primary-antibodies/phospho-ikappab-alpha-ser32-36-5a5-mouse-monoclonal-antibody/9246<br>Anti-IκBα antibody (Cell Signaling, 4812S): https://www.cellsignal.com/products/primary-antibodies/ikappab-alpha-44d4-rabbit-monoclonal-antibody/4812<br>Anti-Vinculin (Cell Signaling, 13901): https://www.cellsignal.com/products/primary-antibodies/vinculin-e1e9v-rabbit-monoclonal-antibody/13901<br>HRP-conjugated goat anti-rabbit IgG(H+L) (ProteinTech, SA00001-2): https://www.ptglab.com/products/HRP-conjugated-Goat-Anti-Rabbit-IgG-H-L-SA00001-2.htm<br>Goat anti-mouse IgG (H + L)-HRP Conjugate (Bio-Rad, 1706516): https://www.bio-rad.com/en-us/sku/1706516-goat-anti-mouse-igg-h-l-hrp-conjugate?ID=1706516<br>Anti-CD31 (BD Biosciences, 550274): https://www.bdbiosciences.com/en-us/products/reagents/flow-cytometry-reagents/research-reagents/single-color-antibodies-ruo/purified-rat-anti-mouse-cd31.550274?tab=product_details<br>Donkey anti-Rat AF647 antibody (Invitrogen, A48272): https://www.thermofisher.com/antibody/product/Donkey-anti-Rat-IgG-H-L-Highly-Cross-Adsorbed-Secondary-Antibody-Polyclonal/A48272TR<br>Donkey anti-Mouse IgG (H+L) Alexa Fluor™ 488 (Invitrogen, A-21202): https://www.thermofisher.com/antibody/product/Donkey-anti-Mouse-IgG-H-L-Highly-Cross-Adsorbed-Secondary-Antibody-Polyclonal/A-21202<br>Donkey anti-Rabbit IgG (H+L) Alexa Fluor™ 546 (Invitrogen, A10040): https://www.thermofisher.com/antibody/product/Donkey-anti-Rabbit-IgG-H-L-Highly-Cross-Adsorbed-Secondary-Antibody-Polyclonal/A10040

Eukaryotic cell lines

Policy information about cell lines and Sex and Gender in Research

Cell line source(s)	HEK293T cells (ATCC, CRL-3216); COS-7 (ATCC, CRL-1651); MDCK (ATCC, CCL-34); U-2 OS (ATCC, HTB-96); B16-F10 (ATCC, CRL-6475); 4T1 (ATCC, CRL-2539); MDA-MB-231 (ATCC, HTB-26); U-87 MG (ATCC, HTB-14)
Authentication	None of the cell lines used were further authenticated.
Mycoplasma contamination	All cells were tested negative for mycoplasma.
Commonly misidentified lines (See ICLAC register)	No commonly misidentified cell lines were used in the study

Animals and other research organisms

Policy information about studies involving animals; ARRIVE guidelines recommended for reporting animal research, and Sex and Gender in Research

Laboratory animals	5-10 weeks CD-1 (Charles River Laboratories, 022), C57BL/6 mice (Jackson Laboratory, 000664), and 4-8 weeks J:Nu (Jackson Laboratory, 007850) were used in this study
Wild animals	No wild animals were used in this study
Reporting on sex	In the xenograft model, female J:Nu immunodeficient mice (Homozygous for Foxn1<nu>) were used in the study. Otherwise, sex was not considered in this study. Mixed male and female mice were used in the remaining study. Mice of different genders were assigned

	randomly to different groups.
Field-collected samples	No field-collected samples were used in this study
Ethics oversight	The Animal Care and Use Committee (ACUC) of Johns Hopkins University approved the animal protocol (MO21E409 and MO24E325) for this study.

Note that full information on the approval of the study protocol must also be provided in the manuscript.

Plants

Seed stocks	N/A
Novel plant genotypes	N/A
Authentication	N/A
